# Patent ductus arteriosus treatment trends and associated morbidities in neonates

**DOI:** 10.1038/s41598-021-89868-z

**Published:** 2021-05-21

**Authors:** Joonsik Park, So J Yoon, Jungho Han, In G Song, Joohee Lim, Jeong E Shin, Ho S Eun, Kook I Park, Min S Park, Soon M Lee

**Affiliations:** Department of Pediatrics, Yonsei University College of Medicine, 211 Eonjuro Gangnamgu, Seoul, 06273 Republic of Korea

**Keywords:** Paediatric research, Congenital heart defects

## Abstract

To evaluate national epidemiologic data on infants treated for patent ductus arteriosus (PDA) in Korea and analyze outcomes associated with different PDA treatments. We retrospectively evaluated data on 12,336 patients diagnosed with PDA (International Classification of Diseases-10 code: Q250) between 2015 and 2018 from the Health Insurance Review and Assessment database. Among them, 1623 patients underwent surgical ligation (code: O1671). We used birth certificate data from Statistics Korea to estimate the prevalence, diagnosis, and treatment of PDA. The prevalence of infants with PDA was 81 infants per 10,000 live births and 45.2% in very low birth weight (VLBW) infants, which increased from 2015 to 2018. PDA ligation was performed in 2571 infants and 22% VLBW infants. Medical treatment was administered to 4202 infants, which decreased significantly, especially in VLBW infants (62% to 53%). The proportion of treatment was as follows: conservative treatment (53.1%), intravenous ibuprofen (24.4%), surgery (20.4%), and oral ibuprofen (10.7%); that among 4854 VLBW infants was as follows: intravenous ibuprofen (46.3%), conservative treatment (33.2%), surgery (22.2%), and oral ibuprofen (14.2%). Surgical treatment had a significantly higher risk (odds ratio 1.36) of mortality than conservative treatment. Surgical and/or medical treatments were associated with a higher risk of morbidity. Recently, increased use of conservative management of PDA has contributed to improved neonatal outcomes in VLBW infants. Select patients may still benefit from surgical ligation following careful consideration.

## Introduction

Patent ductus arteriosus (PDA) occurs in approximately 20-50% of neonates born before 32 weeks gestation and in up to 60% of neonates born before 29 weeks gestation^1,2^. Because of the possibility of spontaneous closure of PDA, the decision whether to treat PDA is still controversial. Treatment options for hemodynamically significant PDA (hsPDA) include conservative management, pharmacologic interventions, surgical ligation, and a transcatheter approach to ductal closure. However, a consensus on PDA management strategies remains elusive. Surgical ligation is usually considered when other medical treatments have either failed or were contraindicated^3^.

Recent studies claimed that surgical ligation of PDA is associated with neonatal mortality, short-term morbidity, retinopathy of prematurity (ROP), bronchopulmonary dysplasia (BPD), and neurodevelopmental impairment in early childhood^4–7^. Other studies indicated that treating infants having the most severe symptoms with the most aggressive PDA treatments could produce bias^8^. There could be confounding effects as suggested by the clinical utility of ligation as a rescue treatment for failed medical or conservative treatment. A meta-analysis showed that surgical ligation of PDA is associated with reduced mortality, but surviving infants are at an increased risk of neurodevelopmental impairment^9^. In current neonatal practice, surgical ligation of PDA is still indicated for infants with large shunts that cause respiratory insufficiency and whose propensity for spontaneous closure appears low^10^. The risks and benefits of surgical ligation for PDA compared with those of conservative treatment are not fully understood^11^.

Prophylactic and early ligation approaches are no longer indicated after a Cochrane Review was published in 2008^12^. The trend in PDA treatment strategy in preterm infants has been moving toward adopting a more conservative approach rather than treating with aggressive methods^13–18^. However, this conservative strategy has limitations when treating high-risk patients who need immediate interventions for PDA even though considering side effects following its treatment. A wide variation in hospital practices was noted, due to an absence of clear evidence of a causal relationship between PDA severity and adverse outcomes to guide management, as well as reports of adverse short- and long-term effects from treatment^19^.

Diverse treatment strategies for PDA are still being used, and national epidemiological data can be useful for comparing medical outcomes following treatment. Thus, this study aimed to evaluate national epidemiologic data on infants treated for PDA in Korea and analyze outcomes associated with different PDA treatments.

## Results

The prevalence of infants diagnosed with PDA was 81 infants per 10,000 live births, and the annual prevalence rate increased from 70 infants per 10,000 live births in 2015 to 94 infants per 10,000 live births in 2018, especially in term infants. The prevalence of PDA diagnosis in very low birth weight (VLBW) infants was 45.2%, which increased by 5.6% over the 4 years studied (Table 1). Among 2571 infants treated with surgical ligation, the prevalence of surgical ligation by year significantly decreased; 4202 infants were treated with medication, and this approach decreased significantly from 2015 to 2018, especially in VLBW infants (62% to 53%). In addition, PDA ligation was performed in 23% of infants with a birth weight of 2,500 g or more, 11% of infants with a birth weight of 1,500 to 2,500 g, and 22% of infants below a birth weight of 1,500 g. The proportion of small for gestational age infants diagnosed with PDA was 4.0% (632 infants) and ranged from 3.8 to 5.9% during the 4 years studied.

**Figure 1:**
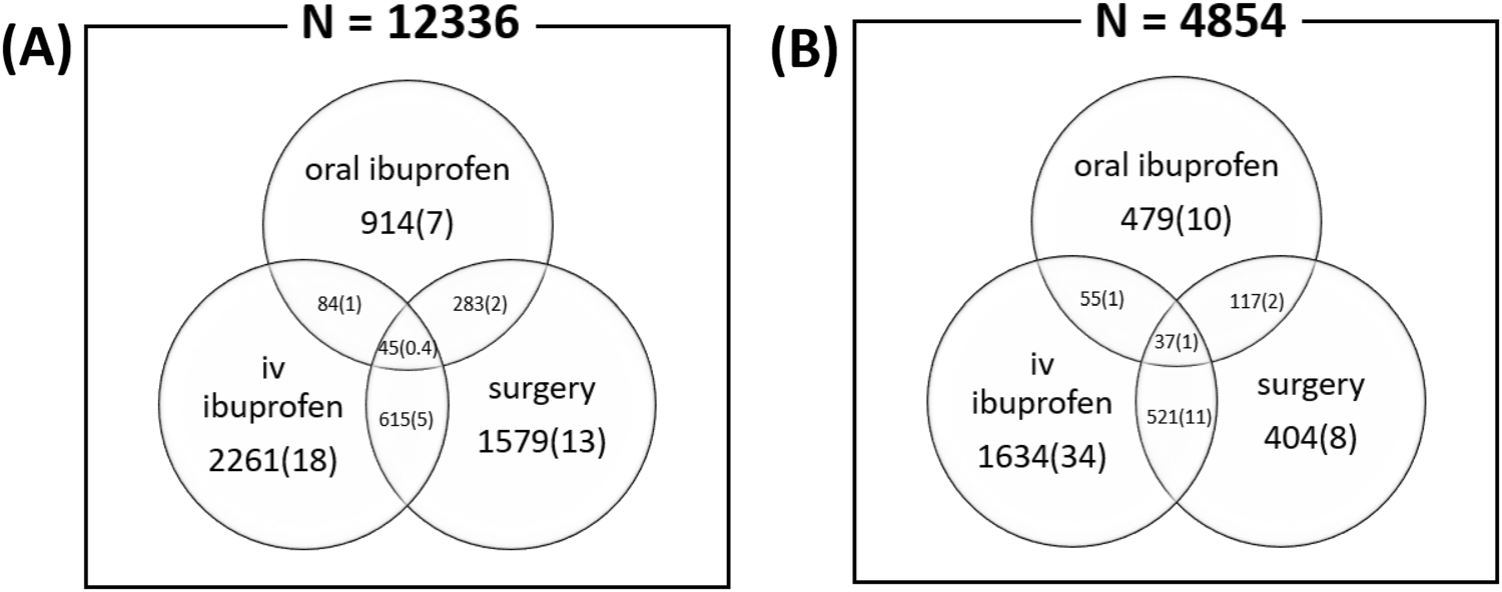
Distribution of PDA treatment strategy among the (**A**) neonate and (**B**) VLBW infants in Korea.

The proportion of various treatment methods used in the entire study group and the VLBW subgroup is shown in Figure 1. Surgery was performed in 2522 infants (20.4%), IV ibuprofen was administered to 2247 infants (24.4%), and 1326 (10.7%) infants were given oral medication. Among 4,854 VLBW infants, 1078 infants (22.2%) underwent surgery, 2247 infants (46.3%) were treated with IV ibuprofen, and oral medication was administered to 688 (14.2%). Combination therapy involving surgery, IV medication, and oral medication was used in 45 infants (0.36%) in total and 37 infants (0.76%) in the VLBW subgroup.

**Figure 2:**
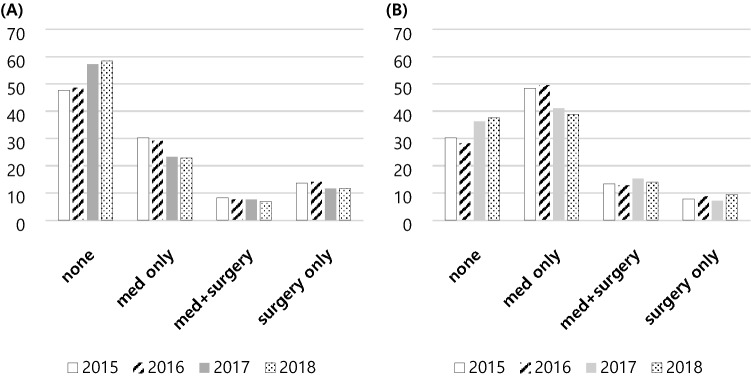
PDA treatment strategy by year among the (**A**) neonate and (**B**) VLBW infants in Korea.

The trends in PDA treatment strategy in Korea by year are shown in Figure 2. A conservative approach was increasingly popular from 2015 to 2018 while the use of medication or surgical treatment decreased. In the VLBW group, the use of conservative treatment also increased, while use of medication alone decreased. However, the percentage of infants undergoing surgical treatment remained similar.

Among patients diagnosed with PDA, the mortality rate was 3.5%, which decreased over the years studied (3.59% in 2015 to 3.55% in 2018, Figure 3). Among infants treated with surgery, the mortality rate was 3.9%, which showed a significantly higher risk (OR 1.36, 95% CI 1.01-1.83, *P*<0.0001) than conservative treatment. The co-morbidities and mortality associated with PDA treatment especially in VLBW infants according to the treatment strategy are shown in Table 2. The surgical and medical treatment groups had a higher risk of morbidity, including BPD, NEC, and sepsis, than the conservative group. However, the risk of IVH was not significantly different compared to that in the conservative treatment group. Medical treatment was associated with an increased risk of ROP compared to the conservative treatment; however, surgical treatment showed a decreased risk of ROP compared to the conservative treatment.

**Table 1:** Prevalence of PDA Diagnosis, surgical ligation, and pharmacological intervention according to the birth weight group.

		2015	2016	2017	2018	Total
Diagnosis of PDA	Number	3,060	3,006	3,213	3,057	12,336
	Total (%)	0.7	0.74	0.9	0.94	0.81
	Preterm (%)	5.69	5.77	6.74	6.74	6.2
	Term (%)	0.33	0.35	0.42	0.45	0.38
	> 2500 g (%)	0.31	0.33	0.4	0.43	0.36
	1500-2500 g (%)	2.27	2.50	3.40	3.32	2.84
	VLBW (%)	41.69	43.69	48.3	47.25	45.02
Surgical treatment of PDA	Number	671	662	621	568	2,521
	Total (%)	21.93	22.02	19.33	18.58	20.44
	> 2500 g (%)	26.37	25.81	20.78	19.01	22.95
	1500-2500 g (%)	12.33	13.28	10.56	8.14	10.95
	VLBW (%)	19.07	21.88	22.50	23.44	21.67
Medical Treatment of PDA	Number	1,182	1,114	995	911	4,202
	Total (%)	38.63	37.06	30.97	29.80	34.06
	> 2500 g (%)	17.71	14.81	10.17	12.09	13.66
	1500-2500 g (%)	34.00	31.31	25.64	24.24	28.43
	VLBW (%)	61.87	62.66	56.46	52.86	58.57

Abbreviations: PDA, patent ductus arteriosus; VLBW, very low birth weight.

(% value is calculated from denominator of total birth in the relevant year).

**Figure 3:**
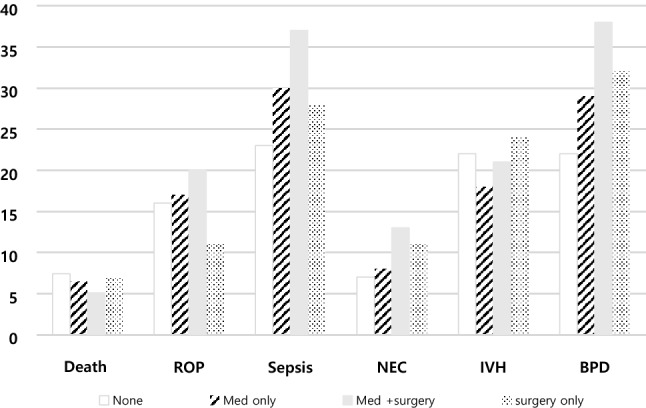
The prevalence of morbidities and mortality according to the treatment strategy by year in VLBW infants.

**Table 2:** Risk analysis for the morbidities according to the treatment strategy.

	None	Med only	Med + surgery	Surgery only	Risk prediction	OR	95% CI
BPD N=1368	353(22)	630(29)	260(39)	125(31)	Med only vs None	1.46	1.25–1.69
					Med + surgery vs None	2.23	1.83–2.71
					Surgery only vs None	1.59	1.25–2.03
IVH N = 982	353(22)	378(17)	148(22)	103(26)	Med only vs None	0.75	0.64–0.88
					Med + surgery vs None	1	0.80–1.24
					Surgery only vs None	1.22	0.94–1.57
NEC N = 421	106(7)	175(8)	96(14)	44(11)	Med only vs None	1.24	0.97–1.60
					Med + surgery vs None	2.35	1.75–3.15
					Surgery only vs None	1.73	1.20–2.51
Sepsis N = 1274	287(18)	643(30)	247(37)	97(24)	Med only vs None	1.94	1.66–2.27
					Med + surgery vs None	2.65	2.17–3.25
					Surgery only vs None	1.45	1.12–1.89
ROP N = 778	237(15)	363(17)	137(20)	41(10)	Med only vs None	1.16	1.01–1.39
					Med + surgery vs None	1.47	1.17–1.86
					Surgery only vs None	0.65	0.46–0.93
Death N = 314	119(7)	134(6)	36(5)	25(6)	Med only vs None	0.82	0.64–1.06
					Med + surgery vs None	0.71	0.48–1.04
					Surgery only vs None	0.83	0.53–1.29

Data are shown as number (%).

Abbreviations: BPD, bronchopulmonary dysplasia; IVH, intraventricular hemorrhage; NEC, necrotizing enterocolitis; ROP, retinopathy of prematurity; Med, medication; OR, odds ratio; SD, standard deviation.

## Discussion

There is still uncertainty and controversy about the management of PDA in preterm infants, resulting in substantial heterogeneity in clinical practice. There has been a shift in recent years from an aggressive PDA closure approach to a more expectant attitude, allowing for spontaneous closure, thus avoiding the need for therapeutic interventions. However, the effect of active treatment compared with that of nonintervention remains unclear. This study evaluated recent PDA treatment trends and identified related outcomes using nationwide population data. Characterizing practice patterns and assessing the relationships between different PDA therapies and their health outcomes may provide useful guidance for identifying the best therapies for treating high-risk infants.

From National Health Insurance data in Korea, the prevalence of PDA in VLBW infants was 45%, while the prevalence of PDA ligation in VLBW infants was 22%, which was comparable to population studies from the US and Canada^4,21,22^. From the healthcare insurance dataset of 429,900 VLBW infants in the US from 1998 to 2015, 35% of infants were diagnosed with PDA and 18% had undergone PDA ligation^22^. From the Canadian Neonatal Network (CNN) database in infants under 32 weeks of gestation, 3,673 infants (25%) were diagnosed with PDA and 26.4% of those infants had undergone PDA ligation in 2012^4^. In accordance with this study, the study from the Korean Neonatal Network, which covers 70% of VLBW infants in Korea^23^, reported that 44% received a PDA diagnosis and 23% underwent ligation, with 27% of primary ligation between 2013 and 2014.

Several studies in recent decades showed that early PDA treatment had no greater benefit for premature infants than alternative supportive strategies^13–18^. In US, PDA ligation peaked at 23.7% in 2004 and decreased to 12.7% in 2015^22^. From the retrospective cohort study of VLBW infants in California, between 2008 and 2014, the annual rate of infants undergoing pharmacologic intervention (31% vs 16%) decreased, whereas the proportion of infants who were not treated (61% vs 78%) increased. This tendency was also found in the current study. The prevalence of treatment in VLBW infants in Korea decreased from 70% to 62% in the VLBW subgroup and that of conservative management increased from 30% to 38% during the study period.

However, for moderate-to-large hsPDA, chances of spontaneous closure are rare, and a combination of severe symptoms can be found, resulting in more invasive treatments for PDA closure^24^. A watchful waiting strategy could not be followed in these preterm infants with severe PDA symptoms and they often required immediate surgical ligation for rescue therapy. In preterm infants less than 28 weeks gestation, 60-70% of the population eventually received medical or surgical therapy for hsPDA^25^. In US, PDA ligation still remained around 12.7% in 2015^22^. From the cohort in California, infants undergoing primary ligation slightly increased from 2008 to 2014. In Korea, around 22% of VLBW infants diagnosed with PDA underwent PDA ligation and a similar trend was observed between 2015 and 2018. Surgical ligation may remain beneficial in certain populations, such as VLBW neonates with unstable vital signs who cannot tolerate conservative managements.

Surgery-related factors may potentially harm infants who undergo ligation^8^. Many studies on surgical ligation have reported adverse outcomes such as increased BPD^26^. Lee et al. retrospectively reviewed data from three neonatal intensive care units and identified long-term complications in infants who underwent PDA ligation including chronic lung disease in 77%, IVH in 39%, NEC in 26%, and ROP in 28%^27^. Our national data showed more complications in the surgical group than in the conservative strategy group, including increased morbidities such as BPD, NEC, and sepsis. In accordance with our study, the CNN has shown a trend of increased morbidities such as BPD, IVH, NEC, and severe ROP related to PDA ligation^4^. However, we assumed that complications were not due to the surgery itself but rather because this population was already at a high risk of complications, as infants usually underwent ligation after a PDA diagnosis. Notably, there may have been survival bias and confounding effects of treatment indication by severity. Patients who underwent treatment for PDA may have been experiencing a more severe medical condition, even after correcting for statistical confounders.

The patients with ROP in our data included those with lower stages of the disease (stages 1 and 2), and no significant difference in ROP was observed between treatment groups. In contrast, another study observed a difference in ROP (>grade 3) in different treatment groups^4^.

Several reports about the safety and feasibility of PDA ligation without associated complications suggest that early surgical ligation minimizes the adverse effects of hsPDA in preterm neonates who are likely to require surgical treatment^28,29^. Some studies have shown that ligation is associated with reduced mortality^4^. However, long-term outcomes remain uncertain due to adverse effects from therapy, higher spontaneous closure rates, and smaller ductal shunts with milder symptoms.

We also observed that ORs for some morbidities such as BPD, NEC, sepsis, and ROP were lower in the “only surgery” group than in the “medication plus surgery” group in Table 2. Interestingly, the CNN study also found more complications with patients who received “both medical and surgical treatment” than “only surgery” group^4^. This implies that for patients with the most threatening hsPDA, it may be more beneficial to initially treat with surgery rather than waiting for failure of the medical treatment. As this trend is only acquired from retrospective data, further randomized controlled investigations are needed.

Our study demonstrated a novel finding that early PDA ligation is superior to PDA ligation after the failure of medical treatment. The timing of PDA ligation can be also important for determining neonatal outcomes^30^. Complications related to PDA ligation can be confounded by poor patient characteristics and thus conclusions should be interpreted with caution. Treatment should be targeted according to the severity of symptoms to reduce adverse effects and less conservative approaches can be justified for select patients.

There are several limitations to this study. There is still no consensus regarding the treatment of PDA and the timing of PDA ligation in Korea. Variations in treatment modalities for preterm PDA between clinical units also exist. Furthermore, as these data were collected retrospectively, causality between treatment and complications cannot be established. Moreover, as the national insurance data rely on only diagnostic codes, detailed medical information was limited.

There could be possibilities of interhospital and interpersonal variation due to limited information that hospital and personal identification codes were de-identified to keep personal information protection.

Transcatheter device closure of the PDA in preterm age has recently gaining its popularity in Korea but it is only limited in few centers. Statistical efficacy of those next generation treatments needs to be answered by future studies.

In conclusion, recent years have shown a trend toward the increased use of conservative management of PDA that has contributed to improved neonatal outcomes in VLBW infants. Nonetheless, according to this data, surgical ligation seems to be beneficial in select patients following careful consideration. Further study targeted at infants requiring surgical intervention will be needed.

## Methods

### Patients and data source

This study included the data on patients diagnosed with PDA (International Classification of Diseases-10 code: Q250) between 2015 and 2018 from their Health Insurance Review and Assessment Service (HIRA) claims. (HIRA dataset no. M20190718866). The HIRA database stores the healthcare claims of almost all Korean residents. Approximately 98% of patients are covered by the National Health Insurance Service while 2% are covered by medical aid^20^. We initially identified 12,336 infants who were diagnosed before 6 months of age. We used birth certificate data from Statistics Korea to estimate the prevalence, diagnosis, and treatment of PDA (https://kosis.kr/statisticsList). The complications associated with PDA included hyaline membrane disease, intraventricular hemorrhage (IVH), BPD, necrotizing enterocolitis (NEC), sepsis, ROP, and death; this information was obtained from the International Classification of Diseases-10 codes inputted by the hospital. Small for gestational age and other information, including gestational age and birth weight, were also obtained from the International Classification of Diseases-10 codes in the HIRA database. Medication data included intravenous ibuprofen (Pedea® Inj.) and oral ibuprofen. The HIRA database contains the sex, region, and payment information for each patient.

### Statistical analyses

The baseline characteristics of the subjects were expressed as means and standard deviations for continuous variables and as percentages for categorical variables. The cohort was stratified according to the gestational age and birth weight or year. Chi-square test was used to compare the neonatal characteristics and complications between the groups. Logistic regression models were used to determine the significant changes in the prevalence of complications, as stratified by the gestational age or birth weight and to obtain odds ratios (ORs) and 95% confidence intervals (CIs) for each risk factor associated with mortality and morbidity in PDA. All statistical analyses were performed using SAS version 9.4 (SAS Institute, Cary, North Carolina). *P*-values <0.05 were considered statistically significant.

### Ethics statement

In this study, all identifiable variables, including claim-, individual-, and organizational-level identification numbers, were re-generated randomly by the HIRA database to protect the patients’ privacy. The study protocol was approved by the Institutional Review Board (IRB) of Gangnam Severance Hospital (IRB No. 3-2020-0147). The need for informed consent was waived owing to the retrospective study design and approved by the same IRB committee.
